# shRNA-Mediated Silencing of Y-Box Binding Protein-1 (YB-1) Suppresses Growth of Neuroblastoma Cell SH-SY5Y *In Vitro* and *In Vivo*

**DOI:** 10.1371/journal.pone.0127224

**Published:** 2015-05-19

**Authors:** Hong Wang, Ruowen Sun, Min Gu, Shuang Li, Bin Zhang, Zuofei Chi, Liangchun Hao

**Affiliations:** Department of Pediatric Hematology/Oncology, Hematology Center, Shengjing Hospital of China Medical University, Shenyang, People’s Republic of China; Sun Yat-sen University Medical School, CHINA

## Abstract

Y-box binding protein-1 (YB-1), a member of cold-shock protein superfamily, has been demonstrated to be associated with tumor malignancy, and is proposed as a prognostic marker in multiple carcinomas. However, the role of YB-1 in neuroblastoma has not been well studied. To investigate the functional role of YB-1 in neuroblastoma, we established a YB-1-silenced neuroblastoma cell strain by inhibiting YB-1 expression using a shRNA knockdown approach. YB-1-silenced neuroblastoma SH-SY5Y cells exhibited a pronounced reduction in cell proliferation and an increased rate of apoptosis *in vitro* and *in vivo* xenograft tumor model. At molecular level, YB-1 silencing resulted in downregulation of Cyclin A, Cyclin D1 and Bcl-2, as well as upregulated levels of Bax, cleaved caspase-3 and cleaved PARP-1. We further demonstrated that YB-1 transcriptionally regulated Cyclin D1 expression by chromatin-immunoprecipitation and luciferase reporter assays. In addition, xenograft tumors derived from neuroblastoma SH-SY5Y cell line were treated with YB-1 shRNA plasmids by intra-tumor injection, and YB-1 targeting effectively inhibited tumor growth and induced cell death. In summary, our findings suggest that YB-1 plays a critical role in neuroblastoma development, and it may serve as a potential target for neuroblastoma therapy.

## Introduction

Neuroblastoma is a childhood tumor malignancy which arises from sympathetic neuroblast cells derived from the neural crest. Neuroblastoma is the most common cancer diagnosed during infancy, and accounts for 7–10% of all childhood cancers and 15% of childhood cancer death [1]. Currently, myeloablative therapy in conjunction with transplantation of autologous bone marrow seems to yield better outcome than conventional therapeutic approaches including surgery, radiotherapy and chemotherapy, yet the survival rate is still unsatisfactory [2,3]. Therefore, understanding the molecular mechanisms underlying the tumorigenesis of neuroblastoma is critical for developing disease-specific targets and novel treatment approaches, thereby improving the survival of neuroblastoma patients. Although a number of gene abnormalities have been demonstrated to be associated with neuroblastoma [1,4], the therapeutic potentials of these genes are yet to be assessed, while novel therapeutic targets remain to be explored.

Y-box binding protein 1 (YB-1) is a member of cold-shock protein superfamily which contain an evolutionally ancient and structurally conserved cold shock domain. YB-1 is a multifunctional protein and plays critical roles in a number of biological processes such as proliferation, differentiation and stress response [5]. As a transcription factor, YB-1 regulates the transcription of a number of genes [6,7], and it is also involved in DNA replication, DNA repair, pre-mRNA silencing and mRNA translation by interacting with other proteins [8–11]. YB-1 has been shown to be overexpressed in tumors of the breast, lung, ovary, colon and prostate [12–16], and such upregulation is highly correlated with tumor progression, invasion, metastasis and angiogenesis [17]. Therefore, the expression level of YB-1 is proposed as a prognostic marker for several types of human cancers [14,18].

Recently, Wachowiak *et al*. reported that YB-1 protein level was elevated in human neuroblastoma tissues and proposed it as a potential tumor marker for neuroblastoma [19]. However, the role of YB-1 in neuroblastoma development is not fully elucidated, and whether YB-1 can serve as a therapeutic target for neuroblastoma remains to be addressed. Hence, the role of YB-1 in neuroblastoma and its therapeutic potential were investigated in this study. Here we show that human neuroblastoma SH-SY5Y cells with stably silenced YB-1 expression by short hairpin RNA (shRNA) strategy exhibited significantly suppressed cell proliferation and induced cell cycle arrest and apoptosis *in vitro*. YB-1 silencing in SH-SY5Y cells resulted in impaired tumorigenicity and delayed tumor onset of xenograft tumors in nude mice. Moreover, xenograft tumors derived from neuroblastoma SH-SY5Y cells were treated with YB-1 shRNA plasmids by intra-tumor injection, and YB-1 targeting effectively blocked tumor growth and induced tumor cell apoptosis. These findings demonstrate that YB-1 plays a critical role in neuroblastoma development, and that YB-1 has potential clinical values in neuroblastoma therapy.

## Materials and Methods

### Cell culture and generation of YB-1-silenced neuroblastoma cell strain

Human neuroblastoma cell lines SH-SY5Y, IMR-32 and SK-N-SH were purchased from the Cell Bank of Chinese Academy of Sciences (Shanghai, China); NGP and SH-EP cell lines were purchased from American Type Culture Collection (Manassas, VA, USA). SH-SY5Y, NGP and SH-EP cells were cultured in DMEM (Gibco, Carlsbad, CA, USA) supplemented with 10% FBS (Hyclone, Logan, UT, USA), while IMR-32 and SK-N-SH cells were cultured in MEM (Gibco) supplemented with 10% FBS. The cells were maintained at 37°C in a humidified atmosphere of 95% air and 5% CO_2_. In order to establish a YB-1-silenced cell strain, short hairpin RNA (shRNA)-encoding oligonucleotides with targeting sequences to YB-1 mRNA was designed as follows: 5’-GATCCCCGTACCTTCGCAGTGTAGGATTCAAGAGATCCTACACTGCGAAGGTACTTTTTAGCTAAAAAGTACCTTCGCAGTGTAGGATCTCTTGAATCCTACACTGCGAAGGTACGGG-3’. The oligonucleotides were cloned into a shRNA expression vector pRNA-H1.1 (GenScript, Piscataway, NJ, USA) (shYB-1), while non-targeting control oligonucleotide fragment was cloned in parallel (shCON). SH-SY5Y cells were plated in 6-well plates and transfected with plasmids (total of 2 μg) using lipofectamin 2000 (Invitrogen, Carlsbad, CA, USA) according to the manufacturer’s protocol. At 24 h post-transfection, cells were cultured in the medium containing 200 μg/ml G418 (Invitrogen) for 2 days and stable cell clones constitutively expressing shYB-1 or shCON (namely SH-shYB-1 and SH-shCON respectively) were selected with 400 μg/ml G418 for over a week. YB-1 expression levels in selected positive clones were determined by real time-PCR, immunofluorescence assay and Western blot analysis.

### RNA isolation and real-time PCR

Total RNA of SH-SY5Y parental cells, YB-1-silenced SH-SY5Y cells (SH-shYB-1) and SH-shCON cells was isolated with RNAsimple Total RNA Kit (TIANGEN Biotech, Beijing, China), followed by reverse transcription using Super M-MLV reverse transcriptase (BioTeke, Beijing, China). Real-time PCR was performed to determine the levels of YB-1 and internal control β-actin using SYBR Green Master Mix (Solarbio, Beijing, China) and respective primers in an Exicycler 96 quantitative fluorescence analyzer (Bioneer, Daejeon, Korea). Primer sequences for YB-1 are: sense, 5’-GTGTTCCAGTTCAAGGCAGTA-3’, antisense, 5’-CGTTCTTTTCCCCACTCTCACTA-3’; primer sequences for β-actin are: sense, 5’-CTTAGTTGCGTTACACCCTTTCTTG-3’, antisense, 5’-CTGTCACCTTCACCGTTCCAGTTT-3’.

### Western blot

Cultured cells were harvested and lysed with NP-40 lysis buffer (Beyotime, Haimen, China) for total protein extraction, whereas xenograft tumor tissues were physically homogenized and lysed with RIPA lysis buffer containing 1% v/v PMSF (Beyotime). Proteins were separated by SDS-PAGE, and transferred onto a PVDF membrane (Millipore, Bedford, MA, USA). The membrane was blocked with 5% milk in TBST buffer (TBS + 0.05% v/v Tween-20), and probed for protein of interest with a specific primary antibody overnight at 4°C, followed by incubation with a secondary antibody for 1 h at room temperature (RT). Anti-YB-1 antibody was purchased from Cell Signaling (Boston, MA, USA), anti-cyclin D, anti-cyclin A, anti-cleaved caspase-3, anti-cleaved PARP, anti-Bcl-2 and anti-Bax antibodies were from Wanleibio (Shenyang, China); HRP-conjugated goat anti-rabbit IgG secondary antibody was purchased from Beyotime. Immune complexes were visualized using the ECL system (Qihai Biotech, Shanghai, China). To verify equal protein loading and transfer, membranes were stripped with stripping buffer (Beyotime) and re-probed with anti-β-actin antibody (Wanleibio).

### Immunofluorescence and Hoechst staining

For immunofluorescence staining, SH-SY5Y, SH-shYB-1 and SH-shCON cells that were cultured on coated glass slides were fixed with 4% paraformaldehyde for 15 min and permeabilized with 0.1% Triton-X 100 (Amresco, Solon, OH, USA) for 30 min at RT. The cells were blocked with goat serum (Solarbio), followed by incubation with anti-YB-1 antibody (1:100, Bioss, Beijing, China) overnight at 4°C and subsequently with Cy-3-conjugated goat anti-rabbit IgG secondary antibody (Beyotime) for 1 h at RT. Cell nuclei were stained with DAPI (Biosharp, Hefei, China), and the slides were mounted and observed under a confocal microscope (Olympus BX53, Japan).

For Hoechst staining, SH-SY5Y, SH-shYB-1 and SH-shCON cells cultured on coated glass slides were fixed and stained with Hoechst staining kit (Beyotime) according to the manufacturer’s instructions. The slides were mounted and observed under an Olympus fluorescence microscope.

### Cell proliferation/MTT assay

SH-SY5Y, SH-shYB-1 and SH-shCON cells were seeded in 96-well microplates at a density of 3 × 10^3^ cells per well, and cell proliferation was assessed by MTT assays at 12 h, 24 h, 48 h, 72 h and 96 h after seeding. At each time point, cells were incubated with culture medium containing 0.2 mg/ml MTT (Sigma-Aldrich, St. Louis, MO, USA) for 4 h in a 37°C incubator. Thereafter, medium was removed and cells were dissolved in 200 μl DMSO per well. The optical density (OD) values at 490 nm, corresponding to the number of viable cells, were read by BioTek microplate reader ELX-800 (BioTek, Winooski, VT, USA). Each assay point was performed in 5 replicates.

### FACS analysis

For cell cycle analysis, SH-SY5Y, SH-shYB-1 and SH-shCON cells were trypsinized, washed with PBS and fixed in 70% ethanol at 4°C. Cells were incubated in propidium iodide (PI) solution containing 50 μg/ml RNase A (Beyotime) for 30 min before analyzed by FACSCalibur flow cytometer (BD Biosciences, Franklin Lakes, NJ, USA). Apoptosis analysis was performed using an Annexin-V-FITC/PI kit according to manufacturer’s protocol (KeyGEN, Nanjing, China). Following staining, cells were examined for apoptotic status by flow cytometry.

### Chromatin immunoprecipitation (ChIP) assay

ChIP assay was performed using an EZ-Zyme chromatin Prep kit and an EZ-ChIP immunoprecipitation kit (both from Millipore) following the manufacturer’s instructions. In brief, SH-SY5Y cells cultured on 150 mm dishes were cross-linked with 1% paraformaldehyde for 10 min at RT, and the reaction was stopped by adding 2.5 ml glycine per plate. DNA-protein complexes were obtained with the kit, and a fraction was withdrawn from the mixture as ‘input’ before immunoprecipitation with anti-YB-1 antibody, anti-RNA polymerase II antibody or mouse IgG (negative binding control). Immunoprecipitated DNA samples as well as the ‘input’ were then subjected to reverse cross-link, RNA and protein digestion and DNA purification. Purified DNA was examined by PCR for the presence of DNA fragments of interest using 2 X Power Taq PCR Master Mix (BioTeke). Two sets of primers were designed targeting different sites on CCND1 (encoding Cyclin D1) promoter region: CCND1p#1 sense, 5’-AATGCACCAAAGAGACA-3’, and antisense, 5’-AAGACCACCGAAGGTTCCTAAT-3’; CCND1p#2 sense, 5’-TGCTTTCTCTGCGCTTCTTG-3’, and antisense, 5’-TGGTTAGCGAGCGTAAAGAGC-3’. Binding of RNA polymerase II on GAPDH promoter was used as a positive control for ChIP experiments.

### Dual luciferase reporter assay

A fraction of CCND1 promoter region containing YB-1 binding motif, namely CCND1p, was cloned into a pGL-3-basic Firefly (FL) luciferase reporter vector (Promega, Madison, WI, USA). SH-SY5Y, SH-shYB-1 and SH-shCON cells were transfected with either pGL-3-CCND1p or pGL-3 vector, plus a Renilla (RL) luciferase reporter vector (pRL-TK) as the internal reference. At 48 h post-transfection, cell lysates were harvested, and FL and RL readings were determined with Luciferase Assay System (Promega) in a Lumat LB9507 luminometer (Berthold, Germany). The activity of the CCND1 promoter was quantified as the ratio of FL/RL which was then normalized to SH-SY5Y cells.

### Colony formation assay

SH-SY5Y, SH-shYB-1 and SH-shCON cells were seeded sparsely at a density of 200 cells per 35 mm dish, and culture medium was changed every three days for about 2 weeks. On the harvest day, colonies were fixed with 4% paraformaldehyde for 20 min at RT, and then stained with Wright’s-Giemsa stain kit (Nanjing Jiancheng Bioengineering Institute, Nanjing, China). Colonies consisting of over 50 cells were counted under an optical microscope. Colony forming ratio was calculated as (colony number/cell number seeded) × 100%.

### Development of human neuroblastoma xenograft tumors in nude mice and shRNA therapy

All animal experiments were conducted with the approval by Experimental Animal Ethics Committee of China Medical University. 10^6^ SH-SY5Y, SH-shYB-1 or SH-shCON cells suspended in 0.2 ml PBS were subcutaneously injected into the right armpit flank of a 8-week-old BALB/c nude mouse (n = 6 each group, Day 0). Tumor volume was measured externally with a caliper every week, and calculated using a modified ellipsoidal equation for determining tumor volume (V): V = [length x (width^2^)]/2 [20]. Mice were cervically dislocated by the end of week 8 post-inoculation and the tumor tissues were excised for detailed examinations.

For shRNA therapy, 10^6^ SH-SY5Y parental cells were subcutaneously injected into the right armpit flank of an 8-week-old BALB/c nude mouse (n = 6 each group). When the tumor sizes reached approximately 100 mm^3^, the mice were randomly assigned to receive a single dose of 20 μg shYB-1 plasmids, shCON plasmids or equal volume of PBS by intra-tumor injection (Day 0). Tumor volume was measured every week since shRNA treatment, and tumors were harvested at week 8 for subsequent experiments.

### H&E staining and TUNEL assay

Excised tumor tissues were fixed in 4% paraformaldehyde, paraffin embedded, and sectioned. Some sections were stained with hematoxylin and eosin (H&E) for histological examination following the standard protocol, and some sections were subjected to TUNEL assay. TUNEL staining was performed with the In Situ Cell Death Detection Kit (Roche, Basel, Switzerland). Sections were permeabilized with 0.1% v/v Triton-X 100 for 8 min at RT. Endogenous peroxidase was blocked by incubating the sections with 2% H_2_O_2_ for 10 min. Sections were incubated with the enzyme solution and TUNEL reaction solution for 1 h at 37°C, followed by incubation with Converter-POD for 30 min at 37°C. Thereafter, Diaminobenzidine (DAB) (Solarbio) was added for a brief incubation of 3 to 5 min followed by counterstaining with hematoxylin for the nuclei. The sections were mounted and observed under an Olympus DP73 optical microscope.

### Statistical analysis

Except for the animal experiments, all the assays were performed in three independent replicates. Raw data were analyzed with GraphPad PRISM software (version 5.0; San Diego, CA, USA). Values are presented as mean ± standard deviation. Comparison between groups was performed using a one-way analysis of variance (ANOVA), and multiple comparisons were performed with Bonferroni post-hoc test. Regardless of statistical test used, mean values were considered statistically different when *p* ≤ 0.05.

## Results

### shRNA-mediated silencing of YB-1 in human neuroblastoma SH-SY5Y cells

**Fig 1:**
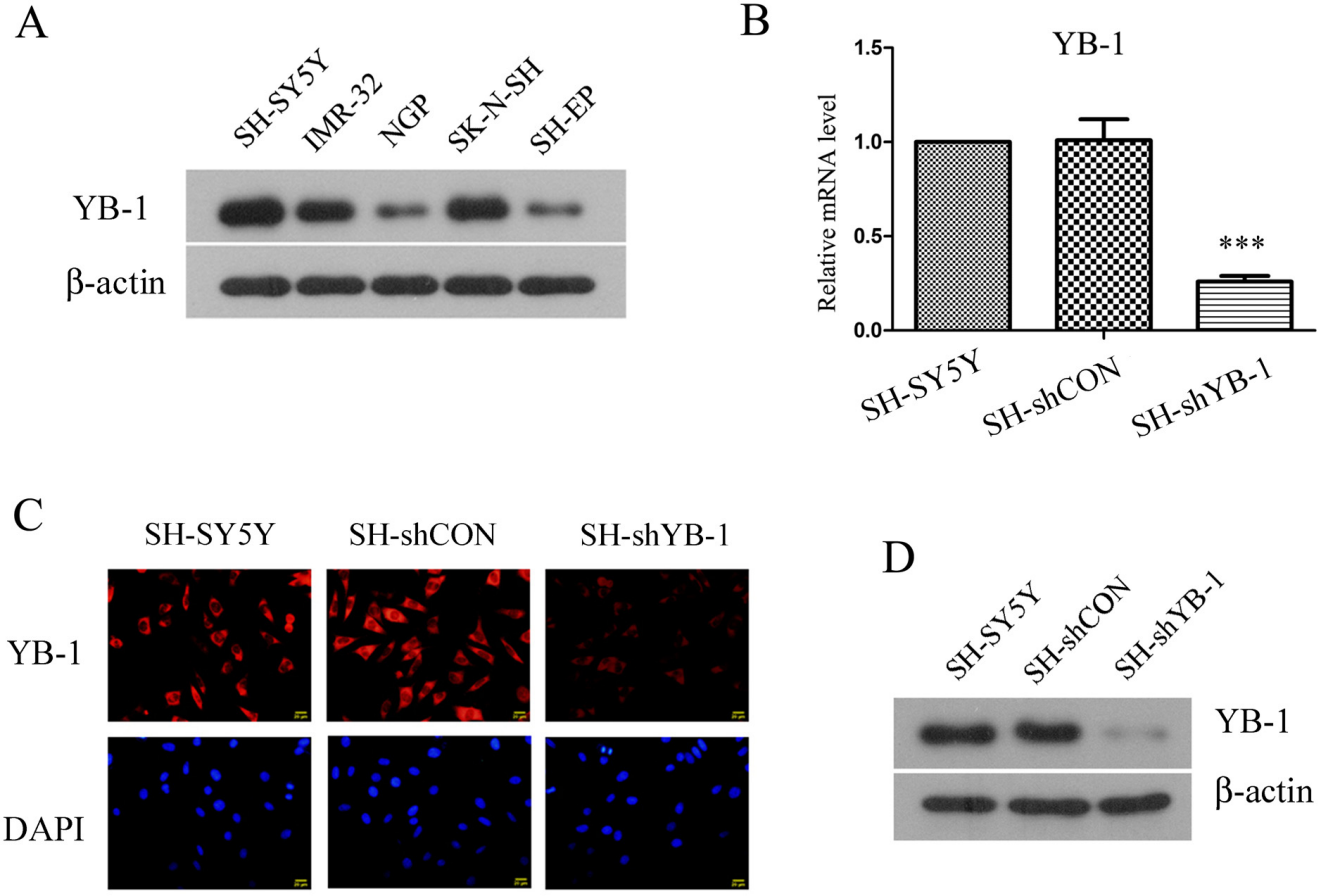
Establishment of YB-1-silenced neuroblastoma cell strain. (A) Expression levels of YB-1 were examined by Western blot analysis in several human neuroblastoma cell lines with β-actin as the internal control. (B-D) SH-SY5Y cells were transfected with YB-1 shRNA construct (SH-shYB-1) or non-targeting control construct (SH-shCON), then selected for stable expression. Cells derived from the positive monoclones were assessed for (B) YB-1 mRNA expression level by real-time PCR, (C) YB-1 protein distribution by immunofluorescence staining and (D) total YB-1 protein level by Western blot analysis. The figure shows the representative images of three independent experiments, and the values are expressed as mean ± standard deviation. Compared with SH-SY5Y control, ***P<0.001.

YB-1 is aberrantly expressed in a variety of tumors, including neuroblastoma [12–16,19]. In this study, we first examined the expression levels of YB-1 protein in five human neuroblastoma cell lines, and found that YB-1 expression varied across the cell lines with the greatest expression in SH-SY5Y cells and relatively lower expression in NGP and SH-EP cells (Fig 1A). Hence, SH-SY5Y cell line with the most abundant YB-1 protein was selected for subsequent experiments. In the pilot experiments, two sets of shRNA constructs against YB-1 mRNA were designed and transfected into SH-SY5Y cells, and both of them suppressed cell proliferation (S1 Fig). The more potent YB-1 shRNA was used to generate YB-1-silenced cell strain (SH-shYB-1) which was established by transfecting SH-SY5Y cells with shYB-1, followed by selection for clones with stable expression. mRNA (Fig 1B) and protein (Fig 1C and 1D) expression levels of YB-1 were knocked down efficiently by YB-1 shRNA in SH-shYB-1 cells, compared with SH-SY5Y parental cells as well as SH-SY5Y cells expressing control shRNA (SH-shCON). Thus, a human neuroblastoma cell strain with stably silenced YB-1 was established.

### Silencing of YB-1 inhibited neuroblastoma cell proliferation

**Fig 2:**
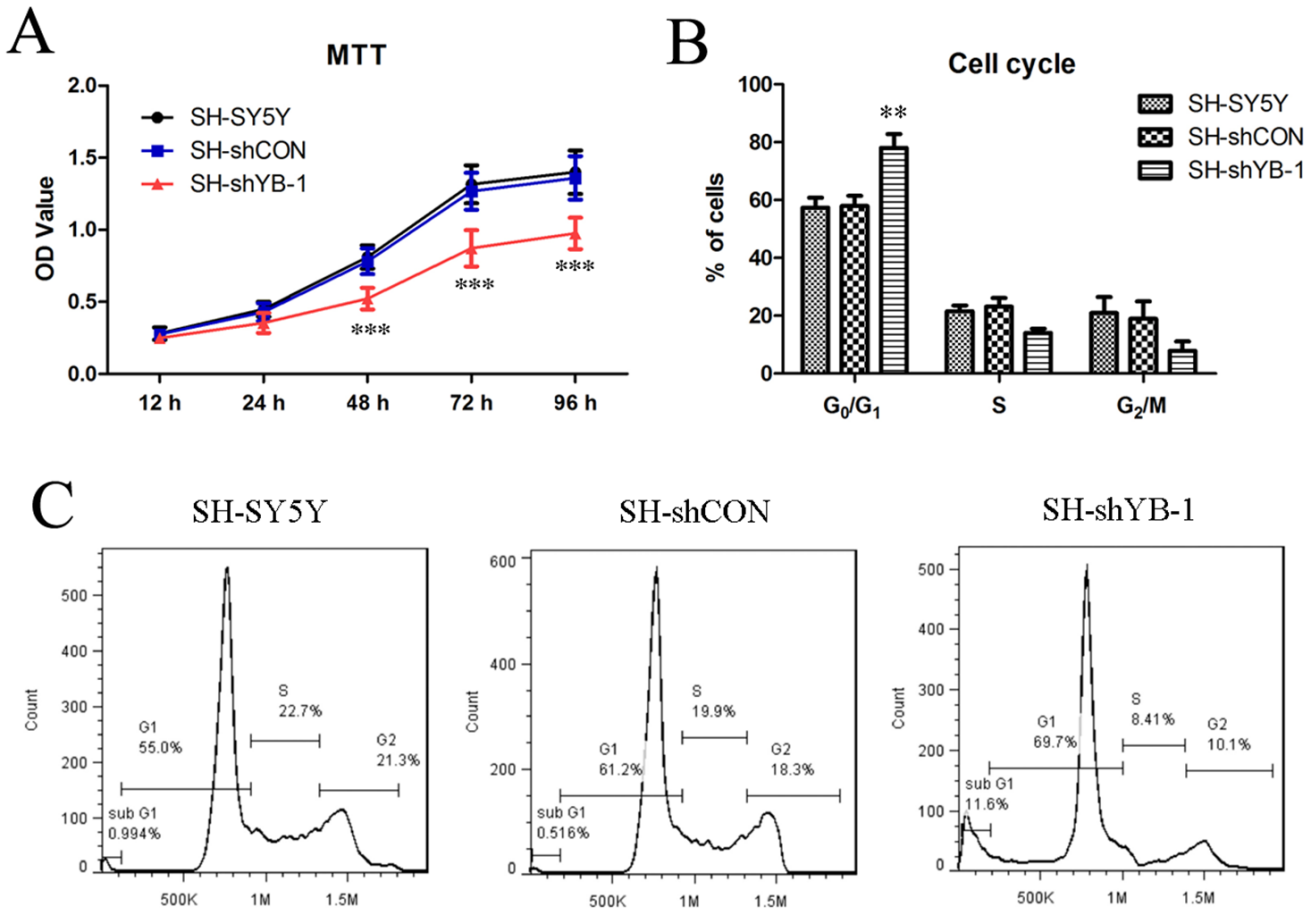
YB-1 silencing suppressed proliferation and induced cell cycle arrest of SH-SY5Y cells. (A) Proliferation of YB-1-silenced SH-SY5Y cells, namely SH-shYB-1, was assessed by MTT assay, in parallel with SH-SY5Y and SH-shCON as control. (B, C) The cells were fixed with 70% ethanol, stained with propidium iodide (PI), and analyzed by flow cytometry for cell cycle stages. B shows the statistical results of cell cycle analysis excluding the apoptotic cells (sub-G_0_/G_1_ peak) and C presents a set of representative FACS data of three independent experiments. Values are expressed as mean ± standard deviation. Compared with SH-SY5Y control, **P<0.01; ***P<0.001.

YB-1 has been demonstrated as a cell cycle stage-specific transcription factor that plays an important role in cell proliferation [6]. Hence, the role of YB-1 in neuroblastoma cell proliferation and cell cycle regulation was investigated in neuroblastoma SH-SY5Y parental and YB-1-silenced cells. Cell proliferation over a time course (12, 24, 48, 72 and 96 h) was assessed by MTT assay, and the results showed a significantly decreased cell proliferation rate of SH-shYB-1 cells wherein YB-1 was silenced, while the control construct shCON showed no effects on cell proliferation (Fig 2A). Moreover, silencing of YB-1 resulted in a marked accumulation of cells in G_0_/G_1_ phase as indicated by cell cycle analysis (Fig 2B and 2C), and such G_0_/G_1_ arrest led to suppression of cell cycle and cell growth. These data suggest that YB-1 plays an important role in regulating cell cycle and cell proliferation of neuroblastoma SH-SY5Y cells.

### YB-1 regulated Cyclin D1 transcription in neuroblastoma cells

**Fig 3:**
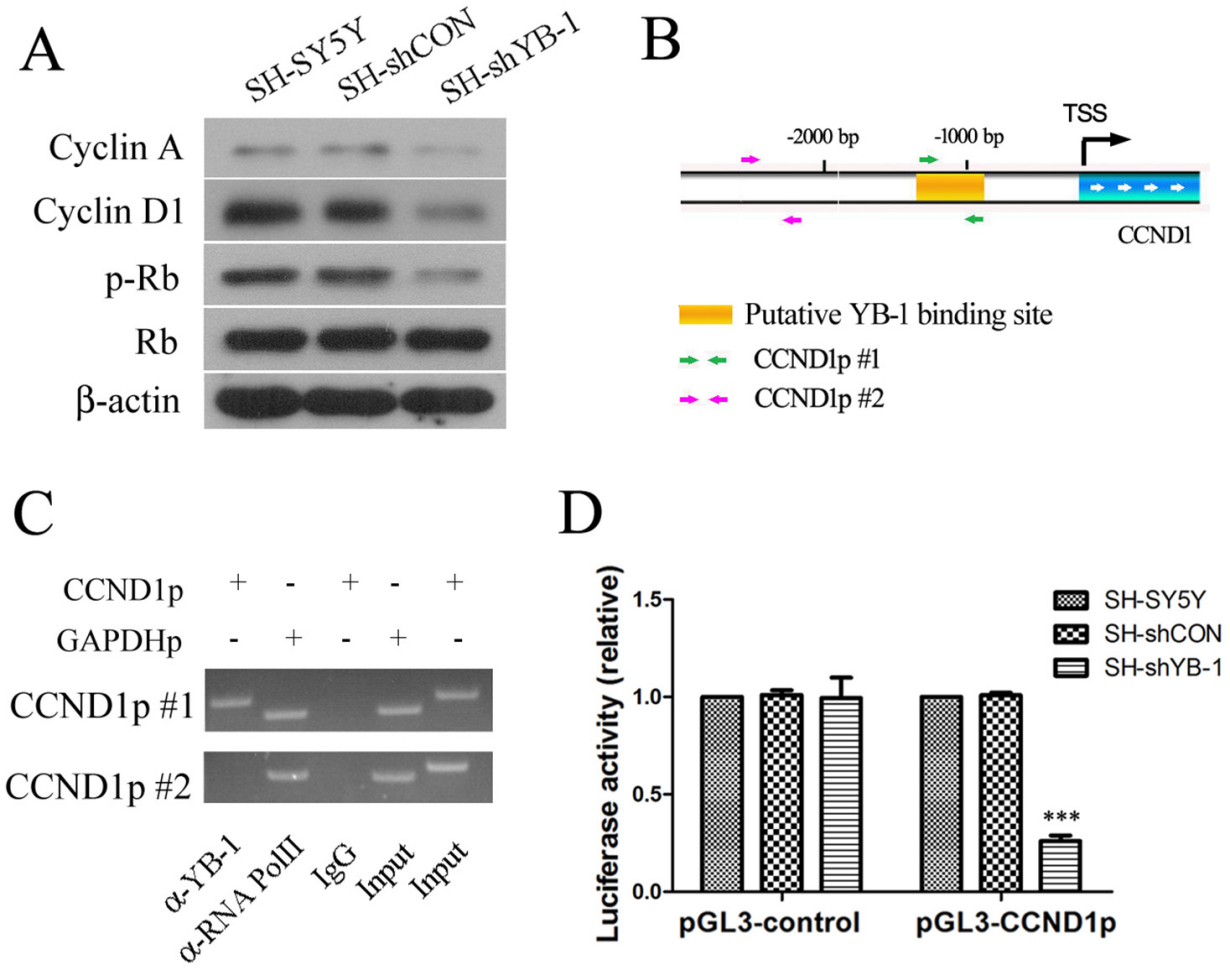
YB-1 regulated Cyclin D1 transcription in neuroblastoma SH-SY5Y cells. (A) Expression levels of cell cycle regulators such as Cyclin A and Cyclin D1 in SH-SY5Y, SH-shCON and SH-shYB-1 cells were examined by Western blot analysis. (B) A schematic illustration of the promoter region of CCND1 which encodes Cyclin D1 indicates the transcription start site (TSS), putative YB-1 binding site and the location of two sets of primers (CCND1p #1 and #2) used for chromatin-immunoprecipitation (ChIP) assay. (C) ChIP assay followed by PCR analysis was performed in SH-SY5Y cells to detect binding of YB-1 on the promoter region of CCND1. Binding of RNA polymerase II (RNA PolII) on GAPDH promoter, which was detected with primers for GAPDH promoter (GAPDHp) by PCR, was used as a positive control for ChIP experiments, whereas IgG served as a negative control for non-specific binding. (D) pGL-3 Firefly luciferase reporter plasmid containing a CCND1 promoter fragment (pGL-3-CCND1) or pGL-3 vector alone was transfected in combination with Renilla luciferase reporter vector pRL-TK into SH-SY5Y, SH-shCON and SH-shYB-1 cells. Luciferase activity representing activity of the promoter was quantified as the ratio of FL/RL which was then normalized to SH-SY5Y cells. Values are expressed as mean ± standard deviation. Compared with SH-SY5Y control, ***P<0.001.

It has been reported that YB-1 upregulation in tumors is associated with elevated levels of a number of cell cycle regulators including Cyclin A and Cyclin D1 [6,21], leading to our hypothesis that transcription factor YB-1 controls cell cycle and cell proliferation by regulating the expression of the cell cycle genes. As shown in Fig 3A, expression of Cyclin D1 was dramatically reduced in YB-1-silenced cells, whereas expression of Cyclin A was decreased to a less extent, probably due to the relative low abundance of the protein. Moreover, downregulation of Cyclin D1 was associated with reduction of phosphorylation of retinoblastoma (Rb) protein in YB-1-silenced cells, which was consistent with the observation in SH-SY5Y cells that were transiently transfected with shYB-1 plasmids (S2A Fig), suggesting that Cyclin D1 may be a target of YB-1 for the regulation of proliferation in SH-SY5Y cells. Later on, ChIP assay was performed to assess the binding of YB-1 on the promoter of CCND1 which encodes Cyclin D1. Two sets of primers, namely CCND1p #1 and CCND1p #2, were designed to target proximal and distal sites on CCND1 promoter region (Fig 3B), and only CCND1p #1-targeted chromatin fragment (-1246 bp to -961 bp) was immunoprecipitated by anti-YB-1 antibody (Fig 3C), indicating that YB-1 bound to CCND1 promoter at around 1000 bp upstream of CCND1 transcription start site. Furthermore, CCND1 promoter region containing YB-1 binding motif was cloned into pGL-3 luciferase reporter vector, which was then transfected into SH-SY5Y, SH-shCON and SH-shYB-1 cells. The effect of YB-1 on the transcription of CCND1 was evaluated by dual luciferase reporter assay (Fig 3D). The results indicated a significant reduction in CCND1 promoter activity as a result of YB-1 silencing, implying that YB-1 is required to facilitate the transcription of CCND1. In addition, the levels of Cyclin D1 were correlated with YB-1 expression level across the tested neuroblastoma cell lines (S2B Fig), suggesting that YB-1 promotes Cyclin D1 expression in neuroblastoma cells. Collectively, the data suggest that YB-1 binds to the promoter of CCND1 and promotes its transcription in neuroblastoma cells in order to modulate cell proliferation.

### Inhibition of YB-1 promoted apoptosis in SH-SY5Y cells

**Fig 4:**
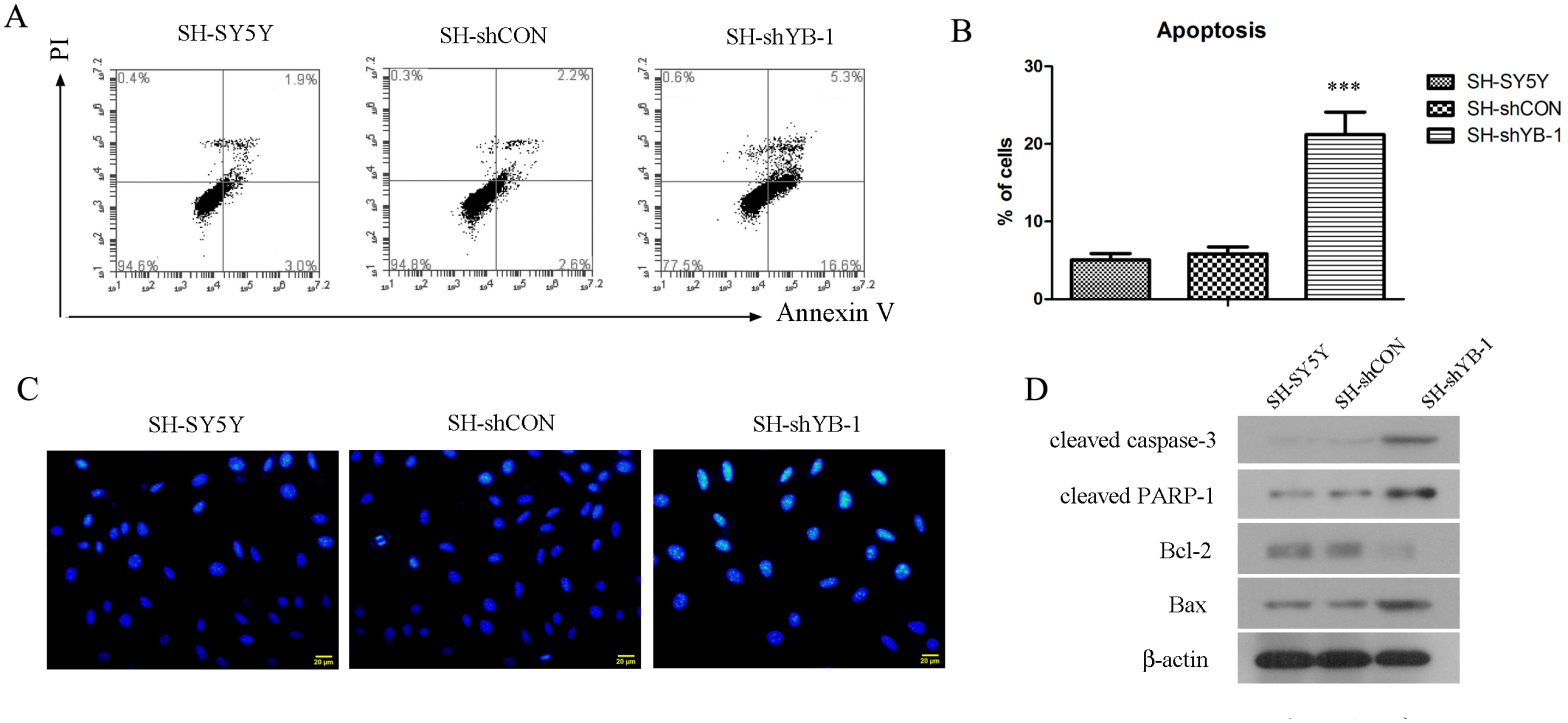
YB-1 silencing induced apoptosis in neuroblastoma cells. (A) SH-SY5Y, SH-shCON and SH-shYB-1 cells were double stained with anti-Annexin-V-FITC antibody and PI, followed by FACS analysis for cell apoptosis. Cells that fell in the lower right quadrant were characterized as early apoptotic cells which were statistically analyzed in B. (C) Cells were incubated with Hoechst stain, whereby the fluorescent stain that was taken by the apoptotic cells bound to DNA and emitted fluorescence under a fluorescent microscope. (D) The levels of several critical apoptosis markers were examined by Western blot analysis with β-actin as the internal control. The figure shows the representative images of three independent experiments, and the values are expressed as mean ± standard deviation. Compared with SH-SY5Y control, ***P<0.001.

YB-1 is also know to exert anti-apoptotic functions in tumors [7,22], thus we further studied the role of YB-1 in the apoptosis of neuroblastoma cells. Early apoptotic cells, which are characterized as Annexin V+ PI- by flow cytometry [23,24], were robustly increased in SH-SY5Y cells with stable or transient knockdown of YB-1, compared with the respective SH-SY5Y and SH-shCON cells (Fig 4A and 4B, S3 Fig). Meanwhile, Hoechst assay was performed to detect DNA fragmentation, which is also an indicator of apoptosis. As shown in Fig 4C, compared with the control cells, the fraction of Hoechst-positive cells were remarkably increased in SH-shYB-1 cells, suggesting that YB-1-silenced cells were more susceptible to apoptosis. Furthermore, the levels of a group of critical apoptosis markers were examined by Western blot analysis. Consistently, the levels of pro-apoptotic proteins such as cleaved caspase-3, cleaved PARP-1 and Bax were elevated in SH-shYB-1 cells, whereas the anti-apoptotic Bcl-2 was downregulated (Fig 4D), indicating a protein profile favoring apoptosis. In summary, YB-1 silencing led to enhanced apoptosis and altered profile of apoptosis-associated proteins in SH-SY5Y cells, suggesting that YB-1 may exert an anti-apoptotic function in neuroblastoma by modulating the levels of these apoptotic proteins.

### YB-1 silencing impaired tumorigenicity and tumor growth of SH-SY5Y cells *in vivo*

**Fig 5:**
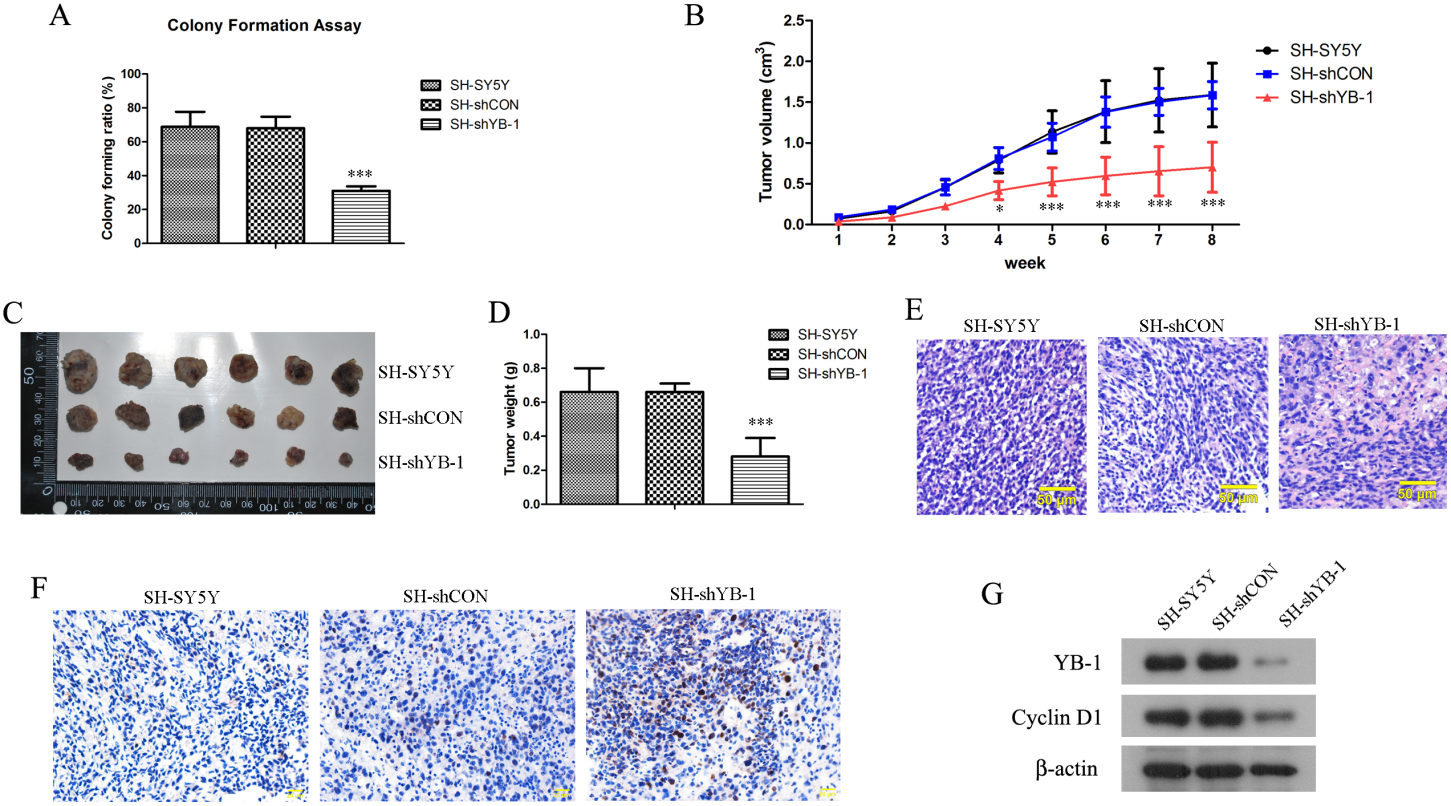
YB-1-silenced SH-SY5Y cells exhibited reduced tumorigenicity and delayed tumor formation *in vivo*. (A) SH-SY5Y, SH-shCON and SH-shYB-1 cells were seeded sparsely on culture dishes and allowed to form colonies for 2 weeks. Colonies consisting of more than 50 cells were counted and colony forming ratio was calculated. (B) 10^6^ SH-SY5Y, SH-shYB-1 or SH-shCON cells were inoculated subcutaneously into an 8-week-old BALB/c nude mouse (n = 6 each group), and tumor volumes were measured externally with a caliper every week and calculated as previously described. (C, D) By end of week 8, the mice were sacrificed, and the tumors were excised, photographed and weighed. (E) Tumor tissues were fixed, paraffin embedded, sectioned and stained with hematoxylin and eosin (H&E) for histological examination. (F) Tumor sections were permeabilized, enzyme inactivated, and incubated sequentially with TUNEL reaction solution, Converter-POD and DAB, followed by counterstaining with hematoxylin. Fragmented DNA was labeled and turned brownish under an optical microscope. (G) Tumor tissues were lysed and total proteins were extracted. Expression levels of YB-1 and Cyclin-D1 in the tumor cells were examined by Western blot analysis. E-G show representative images from all the experimental mice. Values are expressed as mean ± standard deviation. Compared with SH-SY5Y control, *P<0.05; ***P<0.001.

In order to investigate the role of YB-1 in tumorigenesis of neuroblastoma, tumorigenicity of YB-1-silenced SH-SY5Y cells were assessed *in vitro* and *in vivo*. Colony forming capacity is a well-established indicator of tumorigenicity of cancer cells *in vitro* [25]. YB-1 silencing resulted in a significant decrease in the number of colony formed as compared to the control (Fig 5A), implying a reduced tumorigenicity due to YB-1 silencing. When inoculated subcutaneously in nude mice, SH-shYB-1-derived tumors grew more slowly compared with tumors derived from the control cells (Fig 5B–5D), indicating that YB-1 silencing reduced the capacity of SH-SY5Y cells to develop tumors *in vivo*. Histological examination revealed a significant higher frequency of apoptotic events such as nuclear condensation and cytoplasm lysis in the tumors derived from YB-1-silenced cells (Fig 5E), and the overwhelming apoptosis due to YB-1 silencing was confirmed by TUNEL assay (Fig 5F). Furthermore, expression of Cyclin D1 was significantly reduced in SH-shYB-1-derived tumors (Fig 5G), suggesting YB-1-regulated Cyclin D1 expression might be a key mechanism for the tumorigenesis of SH-SY5Y cells *in vivo*.

SH-SY5Y cells are a sub-clone of SK-N-SH cell line derived from the bone marrow biopsy of a neuroblastoma patient, and they express markers typically found in immature neurons and possess the potential to differentiate spontaneously into neurons at a low rate [26]. Pathological analysis of the xenograft tumors based on the Shimada system [27] revealed that all the parental SH-SY5Y-derived tumors (6/6) were in an undifferentiated state; 1 case of poorly differentiated neuroblastoma (< 5% cells with differentiating characteristics and presence of neurofilaments) was found in the xenograft tumors derived from SH-shCON cells, and the rest (5/6) SH-shCON-derived tumors were undifferentiated; in contrast, all the SH-shYB-1-derived tumors (6/6) were poorly differentiated neuroblastomas; no highly differentiated neuroblastoma (> 5% cells with differentiating characteristics, presence of mature ganglion cells and abundant neurofilaments) was found in any group. These results suggest that YB-1 may suppress differentiation of SH-SY5Y cells.

### Targeting YB-1 by intra-tumor shRNA injection inhibited tumor growth in mice

**Fig 6:**
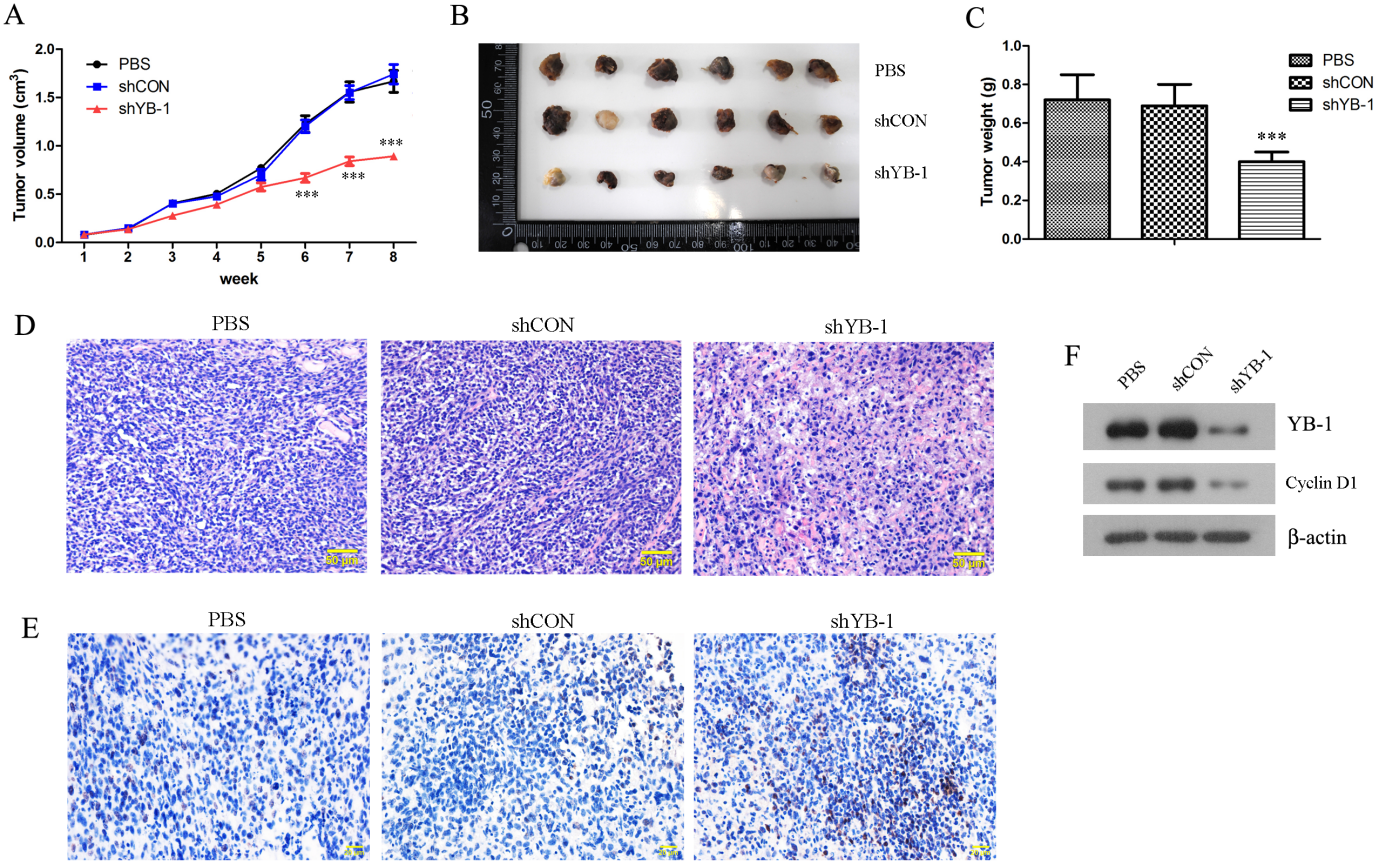
Targeting YB-1 by intra-tumor shRNA injection inhibited tumor growth in mice. SH-SY5Y cells were inoculated subcutaneously to 8-week-old mice at 10^6^ per mouse. When the tumors grew to approximately 100 mm^3^, the mice were randomly assigned to receive an intra-tumor injection of PBS, shCON plasmids or shYB-1 plasmids (n = 6 each group). (A) Tumor volumes were measured every week for 8 weeks from the day of shRNA injection. (B, C) Upon termination of the experiment at post-treatment week 8, the mice were sacrificed, and the tumors were excised, photographed and weighed. (D) Tumor tissues were fixed, paraffin embedded, sectioned and stained with hematoxylin and eosin (H&E) for histological examination. (E) TUNEL assay was performed to detect apoptotic cells in the tumor tissues. (F) Expression levels of YB-1 and Cyclin-D1 in the tumor tissues were assessed by Western blot analysis. D-F show representative images from all the mice examined. Values are expressed as mean ± standard deviation. Compared with PBS control, ***P<0.001.

Since we had observed that YB-1-silenced SH-SY5Y cells exhibited a delayed tumor formation in xenograft tumor model, we further investigated the therapeutic values of YB-1 shRNA in treating neuroblastoma. Nude mice were inoculated subcutaneously with SH-SY5Y cells, and randomly assigned to receive an intra-tumor injection of YB-1 shRNA plasmid namely shYB-1, control shRNA plasmid shCON or PBS alone when the tumors grew up to approximately 100 mm^3^. Tumor sizes were followed up for 8 weeks since the day of shRNA injection, and the tumors were excised for detailed examinations when the mice were sacrificed by week 8. Targeting YB-1 by intra-tumor injection of YB-1 shRNA significantly inhibited tumor growth from post-treatment week 6, and the endpoint tumor sizes were remarkably reduced by YB-1 shRNA treatment (Fig 6A–6C). Histological examination showed marked ongoing apoptosis in the tumors treated with YB-1 shRNA (Fig 6D), which was consistent with the results of TUNEL assay (Fig 6E). In addition, poorly differentiated neuroblastomas were detected in all shYB-1-treated tumors (6/6), in 1 out of 6 shCON-treated tumors and in none of PBS-treated tumors. At molecular level, YB-1 shRNA plasmids were successfully delivered into tumor cells and downregulated the expression of YB-1 and Cyclin D1 (Fig 6F). In summary, targeting YB-1 by intra-tumor injection of YB-1 shRNA plasmids effectively inhibited tumor growth, providing promising evidence for gene therapy using YB-1 as the target for neuroblastoma treatment.

## Discussion

YB-1 overexpression and nuclear translocation has been demonstrated to promote tumor progression and malignant transformation in multiple carcinomas [12–16]. Previous studies have argued that YB-1 is an excellent molecular marker for cancer progression and that it could be a useful target for cancer therapy [17]. Wachowiak et al. reported a high frequency of YB-1 overexpression in human neuroblastoma tissues and proposed YB-1 as a novel maker for neuroblastoma [19]. In addition, Zheng et al. identified YB-1 as a neuroblastoma-associated antigen as YB-1-reactive IgG could be detected in the serum of mice immunized with neuroblastoma AGN2a cells [28], and they further demonstrated that YB-1 immunization combined with Treg depletion could activate specific T-cell response against AGN2a cells [29], suggesting an intriguing role and a potential therapeutic value of YB-1 in neuroblastoma. In the present study, we investigated the role of YB-1 in neuroblastoma by establishing a YB-1-silenced neuroblastoma cell strain using RNA interference approach, and showed that YB-1-silenced neuroblastoma SH-SY5Y cells exhibited reduced proliferation and increased apoptosis *in vitro*. In addition, *in vivo* studies demonstrated a delayed tumor onset and compromised tumor growth capacity of SH-SY5Y-derived xenograft tumors as a result of YB-1 silencing. We further evaluated the potential therapeutic value of YB-1 by injecting YB-1 shRNA plasmids into SH-SY5Y-derived xenograft tumors, and the results suggested a promising potential of gene therapy using YB-1 as the target for neuroblastoma.

Silencing of YB-1 expression in neuroblastoma SH-SY5Y cells led to proliferation suppression and cell cycle arrest, suggesting that YB-1 exerts a positive effect in cell proliferation and cell cycle progression. Cell cycle is known to be a fundamental process of cell proliferation, and it has a great impact on tumorigenesis and tumor progression [30]. Cyclin family proteins including Cyclin A, B, D, E, G and H, which work in concert with cyclin-dependent protein kinases (CDKs), are the key regulators for cell cycle [31,32]. Specifically, Cyclin D-CDK4/6 complexes are known to phosphorylate Rb, leading to dissociation of Rb from E2F and activation of E2F-dependent transcription of a group of components that support DNA replication and cell cycle progression [33]. Cyclins D1 that plays a pivotal role in G1/S phase transition is frequently overexpressed in multiple types of cancers, leading to shortened G1 phase and accelerated tumor progression [34–36]. Here we showed that YB-1 directly bound to CCND1 promoter and promoted the transcription of CCND1, and that YB-1 silencing led to reduction of Cyclin D1 expression and Rb phosphorylation, accompanying the suppression of cell proliferation. These results imply that the regulatory role of YB-1 in cell cycle and cell proliferation of neuroblastoma SH-SY5Y cells was mediated, at least partially, through its regulation on Cyclin D1 expression. YB-1-regulated Cyclin D1 transcription was recently reported in lung cancer cells [37], and re-introduction of Cyclin D1 in YB-1-silenced osteosarcoma cells resumed the proliferative capacity of the cells [38]. These findings suggest that YB-1 plays a critical role in cancer cell proliferation of various cancer types via direct regulation of Cyclin D1 expression. In addition, studies have shown that YB-1 can regulate the expression of components of DNA replication machinery and other Cyclins [6,8], which may synergistically promote cell proliferation and tumor growth in neuroblastoma.

Apoptosis is a well-orchestrated cellular program which is regulated and mediated by a number of proteins. Bcl-2 family proteins such as pro-survival Bcl-2 and pro-apoptotic Bax play important roles in the regulation of intrinsic apoptotic signaling and the ratio of Bax/Bcl-2 seems to determine the sensitivity or resistance to the apoptotic stimuli [39]. Cleavage of caspase-3 is an indicator of activated apoptosis cascade, and the cleaved caspases in turn initiate cell degradation events including cleavage of PARP-1 [39,40]. In our study, silencing of YB-1 resulted in downregulation of Bcl-2, upregulation of Bax and elevated levels of cleaved caspase-3 and cleaved PARP-1, which favored apoptosis to occur. Our study indicates that YB-1 exerts an inhibitory effect on cell apoptosis in neuroblastoma SH-SY5Y cells, and the results are consistent with the findings by other groups in different tumor types [22,41,42]. A few possible mechanisms for YB-1-suppressed apoptosis have been proposed: YB-1 acts as a repressor of transcription of cell death-associated Fas-receptor gene [7], and it also directly modulates the expression and activity of apoptotic regulator p53 [43,44].

Inhibition of tumor growth by shRNA-mediated silencing of YB-1 has been observed in several xenograft tumor models using cell lines of breast cancer, lung cancer and glioblastoma [22,45,46], and our study extends the knowledge to neuroblastoma. Furthermore, we report for the first time that targeting YB-1 by intra-tumor injection of YB-1 shRNA could suppress tumor growth in nude mice. In the present study design, a single injection of YB-1 shRNA plasmids potently inhibited tumor growth in mice, so that a stronger efficacy is expected with multiple doses under tolerable toxicity. In addition to the pro-survival and anti-apoptotic functions in tumor cells, YB-1 has been shown to play critical roles in drug resistance by transactivating drug resistance-associated genes including MDR1, MRP2 and MKNK1 [12,47,48]. Tumor cells with downregulated expression of YB-1 exhibit enhanced sensitivity to irradiation and anti-cancer drugs [42,49], suggesting that targeting YB-1 by RNA interference in combination with chemotherapy and/or radiotherapy may potentiate the therapeutic effects in tumor patients. Currently, the major hurdle for the development of small interfering RNAs (siRNA) as therapeutics is the challenge of delivery, especially the problems of stability and bioavaiability [17], so that advancement in the delivery technologies is the primary task and research focus of siRNA gene therapy [50,51].

In conclusion, YB-1 plays a critical role in cell proliferation, apoptosis and tumorigenesis of neuroblastoma cell line SH-SY5Y. In addition, targeted silencing of YB-1 by shRNA is a potent approach to inhibit tumor growth in mice, and it may have clinical implications in neuroblastoma therapy in the future.

## Supporting Information

S1 Fig Silencing of YB-1 by shRNA suppressed SH-SY5Y proliferation. SH-SY5Y cells were transfected with two different sets of YB-1 shRNA constructs (namely, shYB-1-1 and shYB-1-2) in parallel with the non-targeting control shCON. (A) YB-1 expression levels were determined by Western blot analysis 48 h after transfection. (B) At 6 h post-transfection, the medium was changed and the cells were subjected to MTT proliferation assay.
(TIF)

S2 Fig YB-1 regulates Cyclin D1 expression. (A) Expression levels of Cyclin D1, p-Rb and Rb were examined by Western blot analysis in SH-SY5Y cells that were transfected with shYB-1 or shCON at 48 h post-transfection. (B) Correlated expression of YB-1 and Cyclin D1 in various neuroblastoma cell lines.
(TIF)

S3 Fig Transient knockdown of YB-1 promoted apoptosis in SH-SY5Y cells. SH-SY5Y cells were transfected with two different sets of shYB-1 constructs or shCON, and subjected to apoptosis analysis by FACS 48 h after transfection. Early and late apoptotic cells which were statistically analyzed, and the data are expressed as mean ± standard deviation of three independent experiments. Compared with SH-SY5Y control, *P<0.05; ***P<0.001.
(TIF)
