# Supplementary figures and images for: shRNA-Mediated Silencing of Y-Box Binding Protein-1 (YB-1) Suppresses Growth of Neuroblastoma Cell SH-SY5Y In Vitro and In Vivo
Source: PLoS One. 2015 May 19;10(5):e0127224. doi: 10.1371/journal.pone.0127224 (PMC4438073; doi:10.1371/journal.pone.0127224)

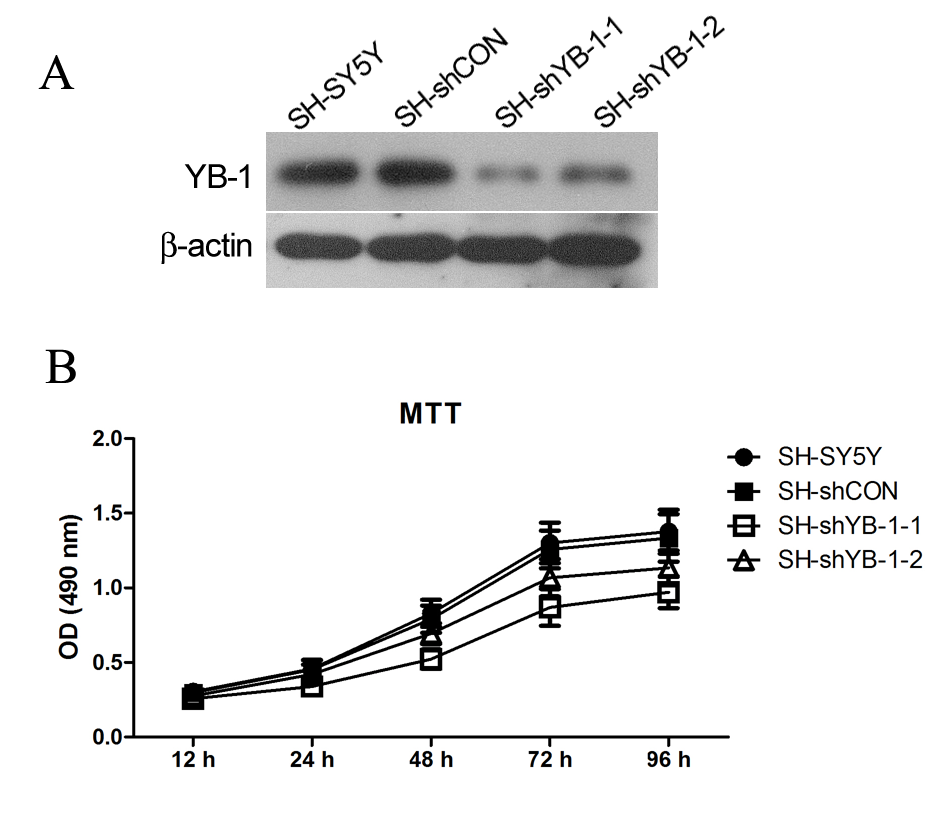

Supplement: S1 Fig — SH-SY5Y cells were transfected with two different sets of YB-1 shRNA constructs (namely, shYB-1-1 and shYB-1-2) in parallel with the non-targeting control shCON. (A) YB-1 expression levels were determined by Western blot analysis 48 h after transfection. (B) At 6 h post-transfection, the medium was changed and the cells were subjected to MTT proliferation assay. (TIF) [file pone.0127224.s001.tif]

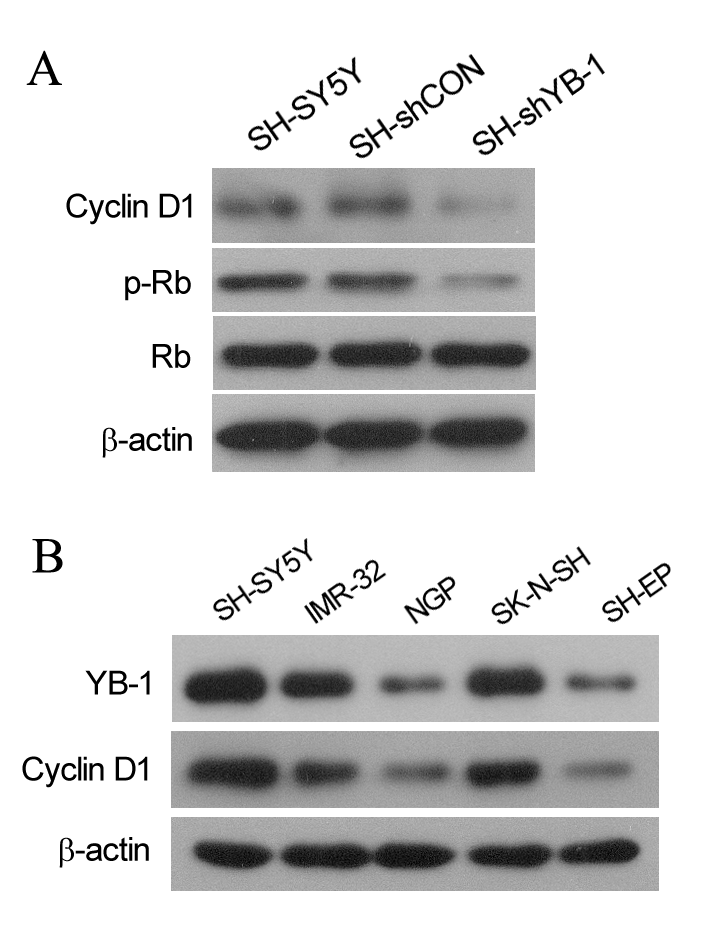

Supplement: S2 Fig — (A) Expression levels of Cyclin D1, p-Rb and Rb were examined by Western blot analysis in SH-SY5Y cells that were transfected with shYB-1 or shCON at 48 h post-transfection. (B) Correlated expression of YB-1 and Cyclin D1 in various neuroblastoma cell lines. (TIF) [file pone.0127224.s002.tif]

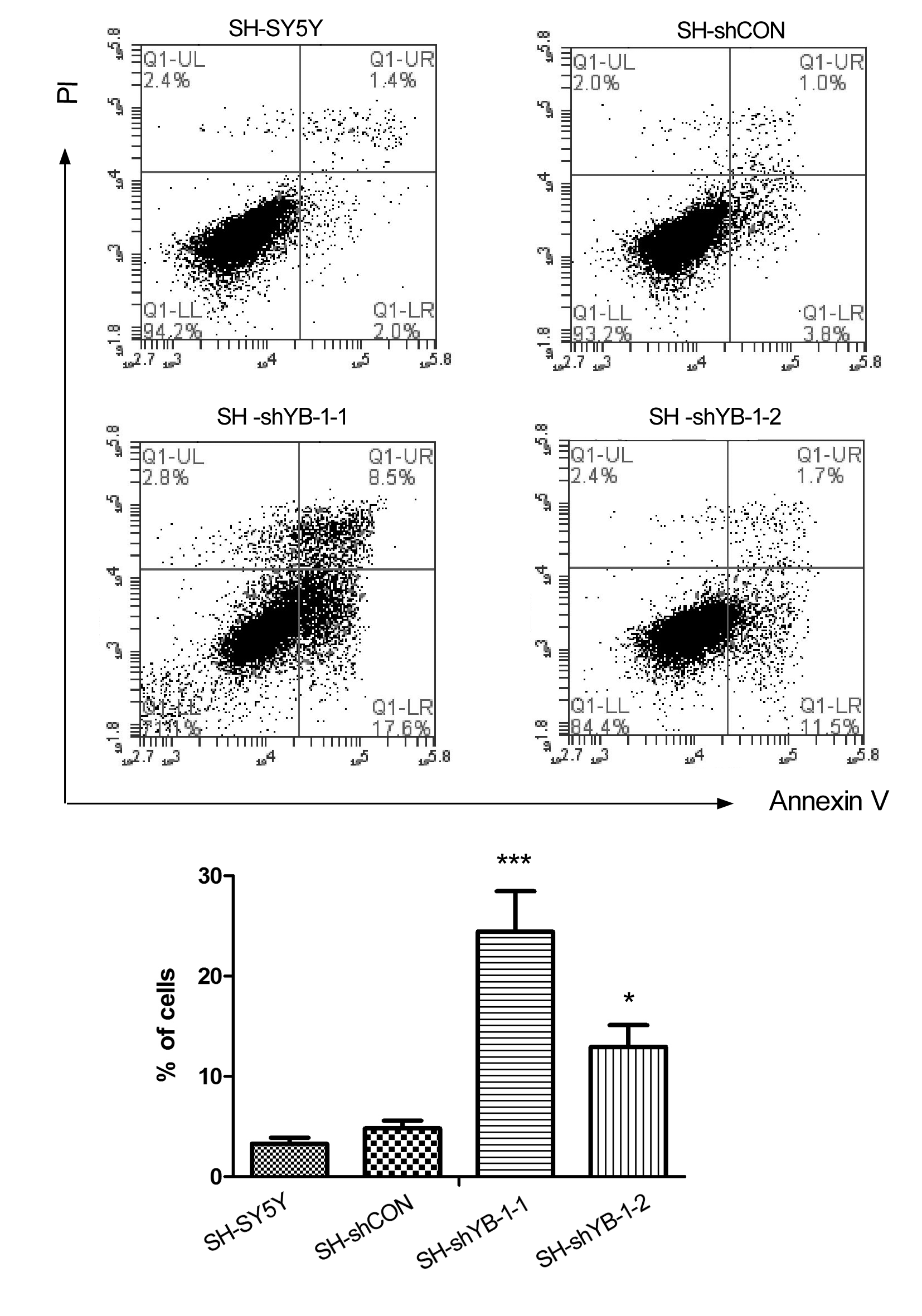

Supplement: S3 Fig — SH-SY5Y cells were transfected with two different sets of shYB-1 constructs or shCON, and subjected to apoptosis analysis by FACS 48 h after transfection. Early and late apoptotic cells which were statistically analyzed, and the data are expressed as mean ± standard deviation of three independent experiments. Compared with SH-SY5Y control, *P<0.05; ***P<0.001. (TIF) [file pone.0127224.s003.tif]
